# CSI-Based Human Activity Recognition Using Multi-Input Multi-Output Autoencoder and Fine-Tuning

**DOI:** 10.3390/s23073591

**Published:** 2023-03-30

**Authors:** Mahnaz Chahoushi, Mohammad Nabati, Reza Asvadi, Seyed Ali Ghorashi

**Affiliations:** 1 Cognitive Telecommunication Research Group, Department of Telecommunications, Faculty of Electrical Engineering, Shahid Beheshti University, Tehran 19839 69411, Iran; 2 Department of Computer Science & Digital Technologies, School of Architecture, Computing and Engineering, University of East London, London E16 2RD, UK

**Keywords:** channel state information (CSI), convolutional autoencoder, human activity recognition (HAR), machine learning (ML)

## Abstract

Wi-Fi-based human activity recognition (HAR) has gained considerable attention recently due to its ease of use and the availability of its infrastructures and sensors. Channel state information (CSI) captures how Wi-Fi signals are transmitted through the environment. Using channel state information of the received signals transmitted from Wi-Fi access points, human activity can be recognized with more accuracy compared with the received signal strength indicator (RSSI). However, in many scenarios and applications, there is a serious limit in the volume of training data because of cost, time, or resource constraints. In this study, multiple deep learning models have been trained for HAR to achieve an acceptable accuracy level while using less training data compared to other machine learning techniques. To do so, a pretrained encoder which is trained using only a limited number of data samples, is utilized for feature extraction. Then, by using fine-tuning, this encoder is utilized in the classifier, which is trained by a fraction of the rest of the data, and the training is continued alongside the rest of the classifier’s layers. Simulation results show that by using only 50% of the training data, there is a 20% improvement compared with the case where the encoder is not used. We also showed that by using an untrainable encoder, an accuracy improvement of 11% using 50% of the training data is achievable with a lower complexity level.

## 1. Introduction

Human activity recognition (HAR) is a vast research field that has attracted significant attention from academic research communities as well as industry players. In the healthcare industry, the effect of using HAR mostly includes the monitoring of the elderly and patients who need constant care, unusual activity detection (e.g., fall detection), age detection, and early detection of diseases such as Alzheimer’s. Data for detecting human activities can be collected via cameras (image and video), wearable/environmental sensors, and smartphones. Vision-based HAR algorithms, despite their high accuracy, have limitations such as violating the privacy, dependence on adequate lighting, and the requirement for direct visibility of the target. As a result, the existence of obstacles or walls can cause problems in data gathering. The use of wearable sensors also may cause discomfort and disturbance in performing activities while still compromising people’s privacy. Collecting the transmitted signal from Wi-Fi access points via smartphones has become a conventional approach that brings advantages over other methods in terms of privacy, availability, ease of installation and use, as well as cost. Two common attributes of the received Wi-Fi signal used in HAR are the received signal strength indicator (RSSI) and channel state information (CSI). RSSI has been vastly used in HAR [1] as well as localization [2] because of its ease of use and acceptable accuracy. Although collecting CSI needs more complex receivers and more processing because it contains both the phase and amplitude information, it is shown to give more accurate results in HAR as compared to RSSI [3,4,5]. CSI is also less sensitive to the distance between Wi-Fi transmitters and receivers and to the obstacles [6].

Machine learning (ML) and, in particular, deep learning (DL) are powerful tools for classification and prediction that have been extensively used to recognize human activity. For example, in [7], Shalaby et al. present techniques using deep-learning-based tools trained by CSI data that are highly accurate in HAR and perform well for high-dimensional and time series data. Autoencoders (AE) are used to extract rich features to enhance classification capabilities. AEs are unsupervised deep learning methods that extract features or reduce the dimensionality of the data, and their applications include denoising, synthetic data generation, feature extraction, etc. The purpose of using AEs, which consist of an encoder and a decoder, is to transfer the input to a space with reduced dimensions and then regenerate the input with maximum similarity to the original one at the output layer. In the encoder, data are compressed, and then in the decoder, the features extracted from the encoder are used to reconstruct the data. Then, the trained encoder can be used in classifiers. In some research papers, AE is used in order to remove the noise [8]. Zou et al. [9] propose a CSI-based method named autoencoder long-term recurrent convolutional network (AE-LRCN) that includes a convolutional neural network (CNN) for feature extraction, a long short-term memory (LSTM) module to reveal inherent temporal dependencies, and an autoencoder to remove noise. Guo et al. [10] propose an LSTM-based encoder and a CNN-based decoder to solve the problem of decreasing accuracy when the classifier is used for different users. To compare the performance of AEs with the rest of the methods for feature extraction, Mihoub et al. [11] use different DL techniques, including AEs, recurrent neural networks (RNN), LSTM, gated recurrent units (GRU), multilayer perceptron (MLP), and random forests (RF) for feature extraction and show that AEs achieve excellent performance in feature extraction.

In [12], a new method based on deep learning is proposed for HAR, which uses CNN-3D combined with convolutional long short-term memory (ConvLSTM) for classifying human behavior in videos. This method is well-suited for real-time HAR applications besides its high performance. There are some limitations to this method, such as the requirement for a large amount of data to obtain high accuracy while limited available training data exists. Moreover, this method is sensitive to noise and missing data. It is worth noting that the requirement of labeled training data for classifier adaptation to each individual is an essential obstacle to the widespread adoption of HAR-based applications. In [13], a multi-resolution fusion convolution network (MRFC-Net) has been proposed to improve the accuracy of recognizing the activities. In [14], a light extraction approach using the residual convolutional network and a recurrent neural network (RCNN-BiGRU) is proposed for optimal feature set selection, and the feature selection is based on the marine predator algorithm (MPA). This method achieves good performance, although the computational cost of this method is high.

In [9], due to the use of AE-LRCN, high performance has been achieved without requiring any expert knowledge, and it is not time-consuming. The proposed method in [10] demonstrates high classification performance compared with KNN, SVM, and RNN. Their method also has improved the stability of activity recognition in different indoor environments. On the one hand, most of the aforementioned HAR techniques need a large amount of data for training, and if the amount of collected data is not high enough, their performance may degrade dramatically. On the other hand, human-centered data collection used for HAR is costly and includes privacy problems (e.g., see [15]). Therefore, it would be very valuable to provide a method that can detect human activities with acceptable accuracy using a small amount of data. To this end, augmented and synthetic data generation methods have been proposed [16]. A generative adversarial network (GAN) is a neural network-based structure specified for creating synthetic data. In simple terms, GAN consists of two components, a generator part and a discriminator part. The generator and discriminator have trained simultaneously, which makes it a challenge to train a stable model based on GAN. If the data available for GAN training are limited, the neural networks used in GAN only generate new repetitive (similar) data from the same limited data and degrade the performance level of the human activity recognition model when facing real and different evaluation data [17]. Although it is common to use GAN when the dataset is limited to generate synthetic data, the two main disadvantages associated with GAN have prompted researchers to explore alternative methods for classification. Furthermore, in reference [18], Prabono et al. proposed a solution to counteract the problems associated with insufficient data by utilizing two autoencoders to extract data features. The first autoencoder is trained with a large dataset. Then, the trained encoder is used by fine-tuning in the second AE to extract more effective features from the smaller dataset. The second AE output is given to SoftMax for classification and labeling. The focus of this study is on data reconstruction, with an emphasis on the outcomes of hyperparameter optimization. The authors stated that the primary challenge in their research was the examination of various feature dimensions to identify the optimal setting for constructing data with higher accuracy. Therefore, automated feature dimension selection could potentially enhance effectiveness in several aspects. Collecting an adequate amount of data poses a significant challenge as an alternative method to address the issue of insufficient data. Moshiri et al. in [19] gather CSI data from human activities such as walking, running, standing, sitting, lying down, falling, and bending, and after converting CSI data into RGB images, feed them to a 2D-CNN layer, and compare the performance with other ML methods.

In this research, to relieve the problem of HAR data gathering, which is a time-consuming and costly task and may include some privacy invasion, a method based on multi-input multi-output convolutional autoencoder (MIMO-AE) and fine-tuning is proposed, which improves the accuracy of the classification by richer feature extraction from a smaller set of training data. The proposed MIMO-AE is based only on convolutional neural networks, which are a well-known choice for processing multidimensional arrays. This network has multiple separate input arrays and hence, multiple separate output arrays. Similar to other autoencoders, this structure has two sub-networks of encoder and decoder, regardless of the number of inputs and outputs. The encoder network fuses two inputs into one array, which is considered an extracted feature. Then, the decoder network tries to reconstruct the two mentioned outputs from extracted features. These outputs are quite similar to the two original inputs. By the end of the training process, the encoder network can be used as the first layer of the classifier. In this approach, the network will serve as a retrainable layer. In other words, by utilizing fine-tuning, this trained network can be retrained simultaneously with other layers of the classifier. Aside from the pretrained encoder, the proposed classifier contains both CNN-based and LSTM-based layers. LSTM cells help the network to learn the sequential aspect of data more efficiently. MIMO-AE has a significant advantage over regular AE in that it requires fewer data. 

The rest of this paper is organized as follows: In Section 2, a brief explanation of CSI, autoencoder, MIMO-AE with its structure, and fine-tuning is provided. Additionally, this section defines the four distinct approaches utilized and gives their corresponding structures. Then, in Section 3, experimental results of the fourth approach and an explanation of comparisons are given. Finally, conclusions are discussed in Section 4. The main contributions of this study can be listed as follows:

1. The use of multi-input multi-output autoencoder for extracting targeted information in CSI data.
2. Modifying models to extract more information from a limited number of samples, and therefore, training models with high accuracy, using small data size.
3. Reducing the complexity of the autoencoder-based CNN HAR models while keeping the accuracy at an acceptable level.

## 2. System Method

In this section, the model used for feature extraction and classification is explained. First, the multi-input multi-output autoencoder is explained. Then, four different approaches for training the model are described in detail.

### 2.1. Channel State Information

Orthogonal frequency-division multiplexing (OFDM) technologies are commonly used in telecommunication networks between each pair of transmitters and receivers to transmit coherent information using the Wi-Fi signal on the channel. In OFDM modulation, messages are superimposed on orthogonal subcarriers and transmitted. Transmitting information using OFDM modulation makes it possible to transmit several signals with overlapping spectra through one channel. To put it another way, in OFDM modulation, a single information stream is split among several closely spaced narrowband subchannel frequencies instead of a single wideband channel frequency. The presence of obstacles causes reflection, scattering, and multipath fading [20]. Therefore, when a person between the transmitter and receiver performs an activity, some changes will be made in transmitting the multipath of the Wi-Fi network. Channel state information (CSI) provides information about the amplitude and phase of the transmitted signals so that we can be aware of changes in Wi-Fi signals, including signal scattering, ambient attenuation, environmental fading (including multipath fading and shadow fading), and power decays because of distance in each transmission path during propagation [21]. Using CSI in this context instead of RSSI has many advantages, including more sustainability, reduced environmental influences, and increased transmission of information [3,22]. In addition, OFDM technology can be used in Wi-Fi devices, and the bandwidth can be divided between several orthogonal subcarriers by using the IEEE 802.11 n/ac standard. It is also possible to use multiple antennas for the transmitter and receiver called multiple input multiple output (MIMO) antennas in the Wi-Fi device, which makes it possible to intensify the multiplexing benefit and reduce channel interference [23]. The CSI data can be represented as a channel matrix:

(1) $$CSI=\left(\begin{array}{ccc} H_{1,1} & \ldots & H_{1,r}\\ \vdots & \ddots & \vdots \\ H_{t,1} & \cdots & H_{t,r} \end{array}\right)$$

where *t* is the number of transmitters, *r* is the number of receivers, and $H_{t,r}$ represents a vector that includes complex pairs of subcarriers. *H* can also be demonstrated as:

(2) $$H_{t,r}=\left[h_{t,r,1},\cdots ,h_{t,r,k}\right]$$

where *k* represents the number of data subcarriers, *h* is a complex number that incorporates the phase and amplitude of CSI. Therefore, each subcarrier can be expressed as:

(3) $$h_{t,r}^{i}=A_{t,r}^{i}e^{j\theta_{t,r}^{i}},i\in \left[1,\ldots ,k\right]$$

In the complex number *h*, *A* is the CSI amplitude, θ is the CSI phase, and *i* is the number of subcarriers in each channel. The number of available subcarriers can also vary according to the type of selected hardware or channel bandwidth. In 20MHZ bandwidth, Raspberry pi4 (Nexmon CSI Tool) can access 56 subcarriers.

Performing an activity or making changes in the environment causes changes in the phase and amplitude. However, the presence of unsynchronized transmitters and receivers can cause random phase offsets in CSI and change it chaotically. In addition, the phase can be influenced by the sampling frequency offset, while CSI usually has an almost fixed range [24]. Therefore, the CSI amplitude is usually used.

### 2.2. Autoencoder

An autoencoder is an unsupervised artificial neural network consisting of an input layer, an output layer, and one or a number of hidden layers. Converting the input space to the hidden space is called encoding, and the inverse is known as decoding. The output of the encoder, which is placed in the latent space, is the extracted features (data with fewer dimensions), which shows the more essential information of the input layer. In this research, AE has been used to extract similar features of examples of a common class so that the performance of the classifier can be improved by using the extracted features. The proposed AE is completely explained in Section 2.3. After learning the encoder in the AE, it is saved and used in the classifier. In this research, the decoder, which is used to reconstruct the input data, is not used in the classifier. The function that is used to make a nonlinear mapping of the *x* input at the encoder is as follows:

(4) $$d_{i}=\sigma (wx_{i}+b)$$

where σ is a nonlinear activation function, $d_{i}$ is encoded features, and *w* and *b* are weight and bias, respectively. The decoder function used to reconstruct the input data is as follows:

(5) $${\hat{d}}_{i}=\sigma (\hat{w}d_{i}+\hat{b})$$

where ${\hat{d}}_{i}$ is the output of the decoder, which is tended to be exactly similar to the input, while $\hat{w}$ and $\hat{b}$ are, respectively, the weight and bias of the decoder.

### 2.3. MIMO-AE

MIMO-AE is an unsupervised neural network that, similar to single-input single-output AE, tries to extract effective features through an encoder so that the output of the decoder can be obtained similarly to the input. MIMO-AE can have different inputs with different natures and essence. Similar to audio-visual tasks [25], which aim to detect emotions, MIMO-AE could receive two inputs with different natures. In audio-visual issues, usually one of the inputs is audio, and the other is an image. Both image data and audio data can be extracted from video data. Generally, inputs in MIMO-AE can also have the same nature. In our proposed research, both inputs are an array of CSI data from the same source. To clarify, they can even have the exact same arrays as input, or we can have repetitive combinations. In [26] Geng et al., similar to the proposed research, MIMO-AE is used for feature extraction from CSI data, which improved the performance compared to previous methods for generating channel charts for user-relative positioning and many other applications. Apart from the nature of the data, MIMO-AE considers the similarities between the inputs as a feature, which is extracted from the encoder. Then, the differences are also stored as weights and biases in the decoder. In the proposed research, only the extracted similarities are considered. As a result, after training MIMO-AE, only the trained encoder of AE is used in the classifier. In order to more easily understand the efficiency of this AE, a very simple example is given.

**Figure 1:**
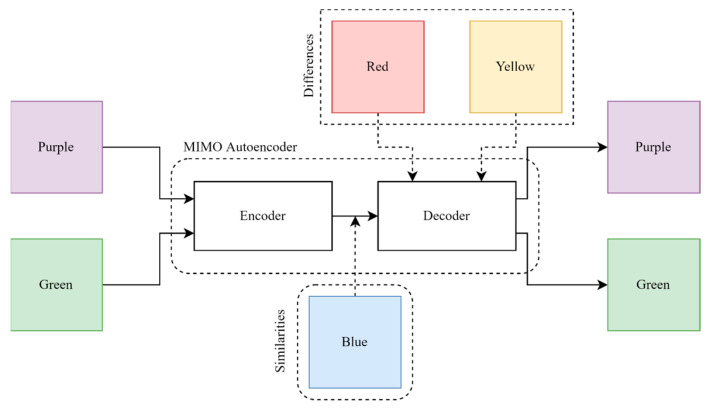
Simple explanation of trained MIMO-AE and relation of inputs and extracted features.

The RGB color images, as depicted in Figure 1, can be considered, one of them displays purple, and the other one shows green. If these two images are considered as inputs to the two-input two-output AE, the extracted feature from the encoder is blue, which is the similarity between these two images. The differences are also red and green, which are stored as weights and biases of the decoder during feature extraction. Finally, red and green combine with the shared color (blue) and reconstruct the inputs at the output.

**Figure 2:**
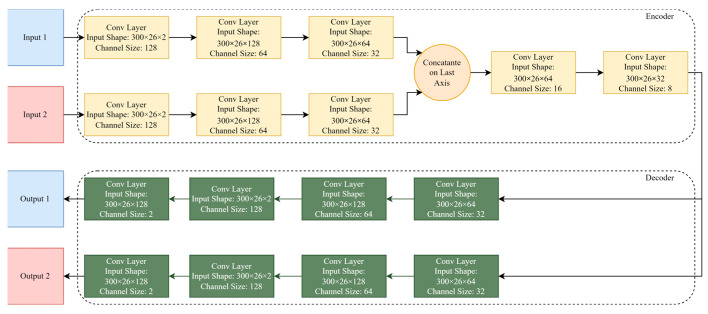
MIMO-AE structure used in this paper, including array sizes and selected hyperparameters and functions.

In the proposed model, a percentage of the data is randomly selected for the training process of AE. Then, samples of classes are compared with each other pairwise, and the extracted similarities are considered as the output of the encoder. It should be noted that two duplicated samples could also be compared. Three-dimensional samples, according to Figure 2, are considered as input for convolutional layers. Then, the features extracted from both inputs through convolutional layers will be merged and again will be passed through the convolutional layers. The encoder’s output is the features made of similar aspects of input arrays. Meanwhile, all the inputs that are from the same class of activity, despite their differences, share similarities regarding their type of activity. Then, the trained encoder is saved and used in the classifier. The decoder is a mirror of the encoder, which consists of several convolutional layers with different channel sizes. All convolutional layer filters are depicted in Figure 2. Finally, the number of channels is adjusted such that the number of channels in input and output is the same at the end.

### 2.4. Fine-Tuning

**Figure 3:**
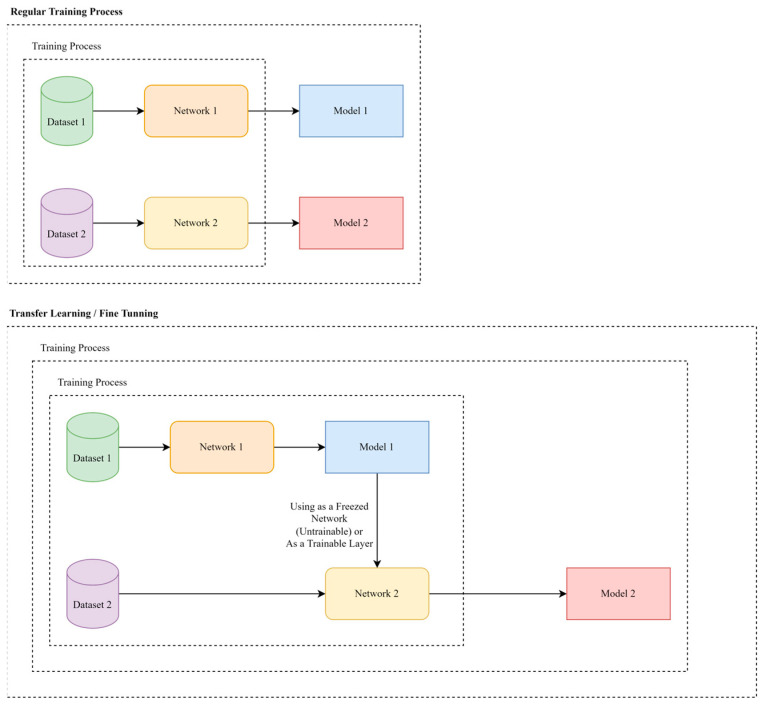
General concept of transfer learning/fine-tuning, based on using pretrained models in the training process of a secondary model.

Transfer learning (TL) is a machine learning method that can improve the performance of a second model by using the pretrained model (with specified weights and biases) as a base point for the second similar model. The TL method has been used in HAR problems in recent years. Hernandez et al. [27] have reviewed the research on TL in HAR. Fine-tuning is also one of the methods based on transfer training. In the fine-tuning method, after training the first model, it is used in another different model related to the first model. Choosing the used part of the first model in the other model is a sensitive step. This choice should be made so that the output of the first model improves the final results of the second model. For example, in Figure 3, after training the first network with dataset 1, model 1 is prepared to be used in another network. Then, network 2, which includes trained model 1, is trained with dataset 2. It should be noted that model 1 in network 2 can be trained again. For instance, in the proposed method, the encoder is the only part of AE which is used in the classifier.

Another difference between the fine-tuning method and TL is in the selected dataset for training the first and second models. In TL, the dataset used to train the first model must be completely different from the dataset used to train the second model. While in the fine-tuning method, if supposedly 10% of the dataset is used for training the first model, the remaining 90% of the same dataset should be used for training the second model. In both methods, after training the first model and determining the weights and biases, in the second model, the weights and biases of the first model will have more imperceptible changes than when they were trained from the beginning. In this research, fine-tuning is used because only a small percentage of the data was used for training the AE model, and a fraction of the remaining data was randomly selected for training the classifier. The data used for AE training do not play a role in training the classifier.

### 2.5. Classifier Included by the Encoder

As mentioned earlier, the main goal of this study is to improve the performance of HAR models in case of extremely limited data using prior knowledge. To do so, the idea of utilizing a two-input two-output autoencoder is suggested. After the training of the autoencoder with a small number of samples, the encoder part of the network will be used as the first layer of the HAR classifier. In order to implement this structure, two different approaches can be selected. In the first approach, the encoder layer can be used as an untrainable layer, or in other words, as a function. This idea causes the achievement of a more capable model while spending almost the same computational cost as the basic HAR classifier. In the second approach, the encoder layer in the advanced HAR classifier would be retrained, or in other words, would be fine-tuned. This model achieves the best recognition results; however, the computational cost is quite higher, considering the fact that the size of the trainable network would be much larger. In this matter, it has been demonstrated through early trials that are incorporating a retrainable encoder as the primary layer of an advanced HAR classifier results in achieving the highest level of performance. In order to justify its computational cost, it is essential to demonstrate that the superior performance of the network is not solely due to its larger structure or the presence of more trainable parameters [28]. Please note that when an encoder layer is added to the classifier, and it requires retraining, the number of trainable parameters will increase, despite the increase in performance. However, if used as an untrainable function, it can increase efficiency. Although using the untrainable encoder results in lower accuracy, the computational cost is significantly reduced. To achieve this, a completely similar structure, compared to the HAR network with a trainable encoder, is used for comparison, while its first layer, i.e., the structure of the encoder is completely untrained. During these simulations, it was observed that this network failed to achieve the recognition accuracy of the targeted HAR classifier network that contained a retrainable encoder subnetwork. The process of trial and error led to the decision to study the selected model in four distinct ways, namely: a simple classifier, a classifier with an untrained encoder, a classifier with an untrainable encoder, and lastly, a classifier with a retrainable encoder. In [19], the whole existing data are used to achieve high performance, and each presented method is based on using just one type of neural network. For example, the recognition is achieved by using just the CNN-2D since the authors believed they could achieve better performance compared to using LSTM. However, in this research, to classify the samples, we use CNN-2D alongside LSTM. Moreover, in the proposed method, because of using the presented encoder in the classifier, the model will be capable of extracting more effective features compared with simple CNN-2D used in [19], and by these features, better performance will be achieved, alongside using less training data.

#### 2.5.1. Model a: Designed Classifier

**Figure 4:**
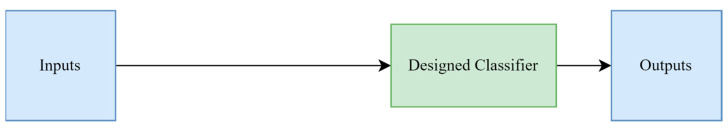
A simple presentation of the classifier without using any pretrained layer.

The first method used in this research is a classifier whose input is a CSI time series. According to Figure 4, the input enters the classifier consisting of convolutional and LSTM layers. 

**Figure 5:**
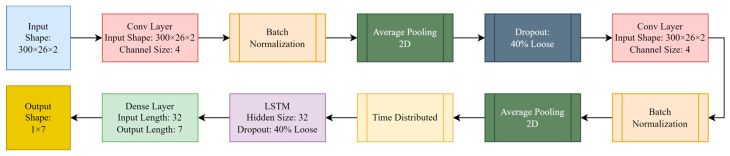
Classifier structure used in this paper, including array sizes and selected hyperparameters and functions.

In this method, the encoder is not used. Sequentially, all the layers used for feature extraction and classification are shown in detail in Figure 5. The classification is performed by the fully connected layer. A sliding window of length 300 slides over the CSI samples, converting the CSI vectors into two-dimensional arrays. Then, a number of samples are randomly selected from the beginning to test and obtain the accuracy of the model, and the rest are used for training and validation. According to the purpose of the research, which is to use fewer samples for training, different amounts of training samples are used for training the model. For feature extraction in this model, we consider the CSI samples, which are stored as arrays of size 300 × 26 × 2 after representation by using a sliding window as input for the convolutional layers shown in Figure 5.

A convolutional neural network is a neural structure that works well for feature extraction and classification of multidimensional data. In this research, this neural network includes several convolutional layers with an activation function, an integration layer, and a fully connected layer. Convolutional layers consist of filters that are applied to multidimensional arrays and perform feature extraction from the data. In this model, a regularizer is applied to CNN layers. In this process, the normalized set of parameters under training will be added to the cost function. The purpose of this work is to minimize this added value using the optimization algorithm. The reason for choosing this approach is that it is one of the first examples of overfitting in the model of exponential enlargement of parameters under training. Keeping these values small through regularization can prevent overfitting. Batch normalization has been used to normalize the variance and mean of each batch or stabilize the distribution of the activation value in the training process. Therefore, the use of batch normalization prevents overfitting and also increases the speed of training. Average pooling 2D has been used almost after each convolution layer. Average pooling 2D decreases the computations by reducing the dimensions of feature maps, excluding channel size. This layer computes the average value of each batch of the feature map that the filter is sliding on. Then, by using dropout in the targeted layer, a certain percentage of neural network neurons are randomly selected and will not be trained at the end of the training step.

According to Figure 5, after the convolutional layers, the LSTM neural network was used. LSTM is a type of RNN in which the problem of forgetting is solved. This idea was first proposed by Hochreiter et al. [29]. This network is capable of learning long-term dependencies, especially in sequence prediction problems. Therefore, this network performs feature extraction well in the field of HAR for prediction or classification using CSI data. Considering that the output format of CNN is different from the input format of LSTM, we will use the time distributed input.

**Figure 6:**
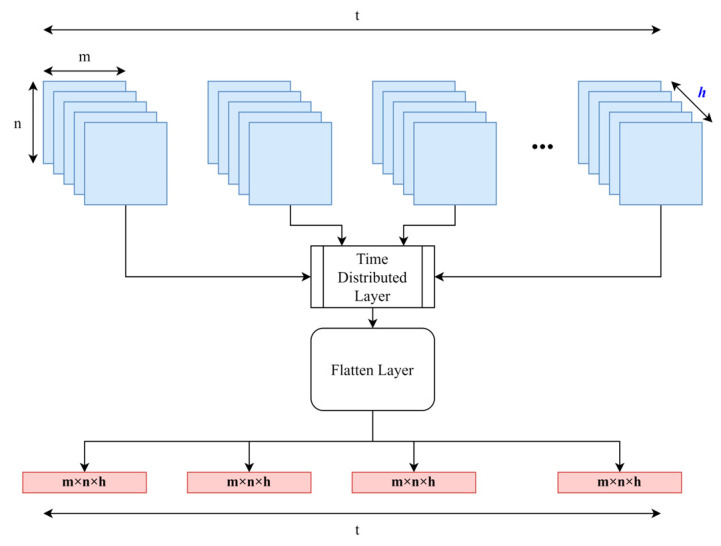
Description of time distributed function, specialized for keeping the order in data.

Time distributed applies a linearization function to the signal while maintaining a time integration. After changing the data representation, it will be used as input for LSTM [30]. As is shown in Figure 6, the size of the multidimensional arrays changes after passing through this function. In simple terms, this function applies the selected layer (here, the flatten layer) to each member of the sequential series. This approach helps maintain the sequential aspect of prior output while changing the shape and size of each member, using the posterior selected layer. In Figure 6, t is the number of input arrays, m and n are the array’s x and y axis, and h is the channel size.

**Figure 7:**
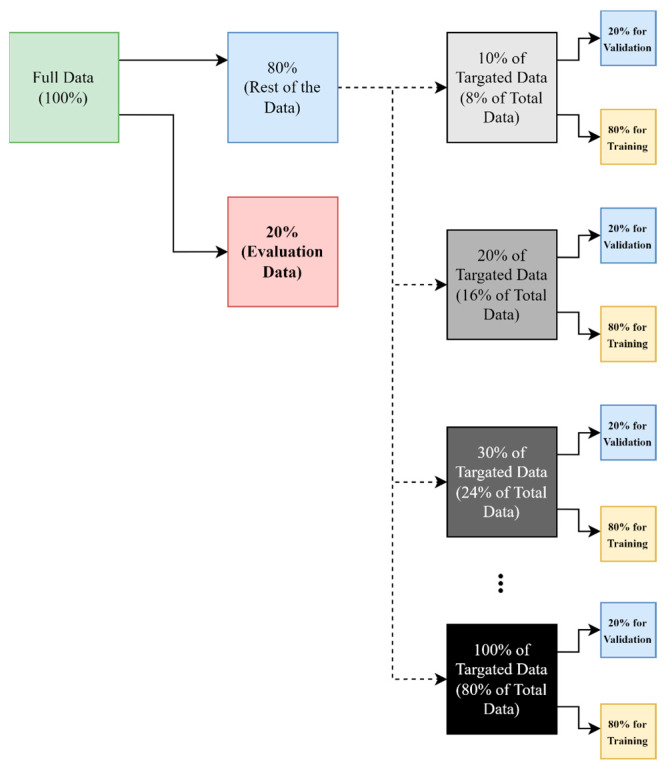
Randomly splitting process of selected dataset, for creating the training, validation, and test (evaluation) datasets.

Finally, the extracted features are entered into the fully connected layer for classification. In the dense layer, seven activity classes are defined using the SoftMax activation function, and the classification is performed. The same approach is implemented in all methods for random split-of-dataset, which is presented in Figure 7.

#### 2.5.2. Model b: Retrainable Encoder

**Figure 8:**
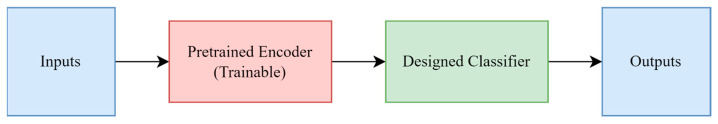
A simple presentation of the classifier included by the retrainable encoder, which is the only difference, compared to Figure 4.

In this method, fine-tuning is used, which is mentioned as one of the methods based on transfer training. The TL method has been used by different researchers to improve the performance of the model in the field of HAR [21,31]. In this method, the AE model is trained by a small percentage of data, then the trained encoder, by using the fine-tuning method, is used in the classifier. In other words, the trained encoder is considered the first part of the classifier. In addition, the encoder can be retrained alongside the classifier. This idea is illustrated in Figure 8. This means that the encoder weights and biases are updated using the information obtained from the new data in the classifier. All three suggested networks share multiple layers (original classifier) after the position of the encoder in their structure.

#### 2.5.3. Model c: Untrainable Encoder

**Figure 9:**
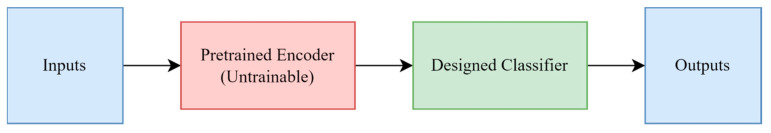
A simple presentation of the classifier included by the untrainable encoder, which is the only difference, compared to Figure 4.

In this method, another model for feature extraction has been implemented on the CSI dataset before classification. In Section 2.3, the MIMO-AE used is explained. After training AE, the trained encoder has been stored. Then, the trained encoder was used as a function or untrainable layer in the classifier, as shown in Figure 9. Based on the definition of fine-tuning in Section 2.4, the use of the previously trained network (here, encoder) in the new model is the main concept of fine-tuning. The preprocessed CSI signal will be used as the input of the model. Then, a pretrained encoder is used as the first layer of the classifier. Note that the encoder will not be retrainable in the classifier. Then, the features extracted from the encoder enter the next layer of the classifier. The difference between this method and the previous one is that the encoder will not be trained again in the classifier.

#### 2.5.4. Model d: Untrained Encoder

**Figure 10:**
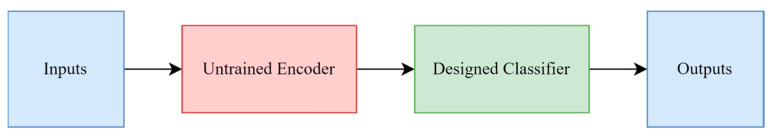
A simple presentation of classifier included by the untrained encoder, which is the only difference, compared to Figure 4.

In this method, an untrained encoder is used. To put it in another way, all the layers used in MIMO-AE are fully depicted in Figure 2. In this case, the untrained structure of the encoder is used as the first layers of the classifier, as depicted in Figure 10. As a result, there is no need to train AE and upload its encoder in this method.

**Figure 11:**
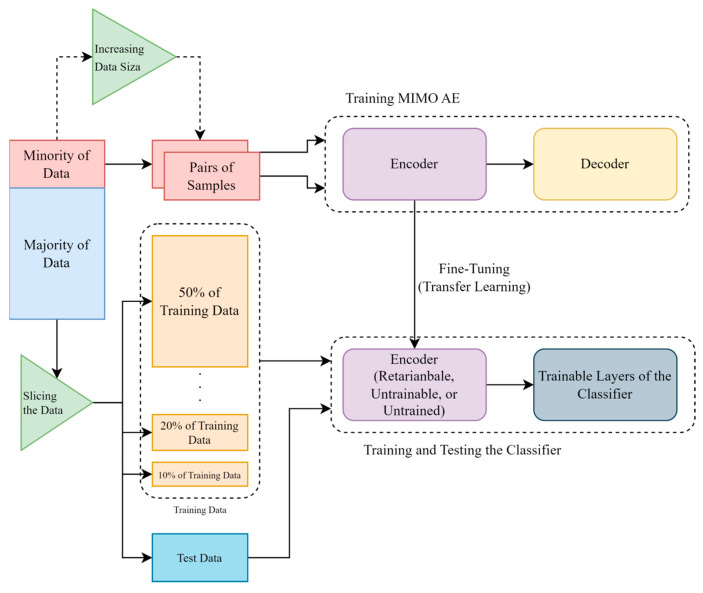
A block diagram of the proposed methods.

In Figure 11, first, the dataset is split. A minor percentage of the dataset will be divided to train the autoencoder in order to use its trained encoder in the classifier by the fine-tuning method. In the multi-input multi-output autoencoder’s training process, the data have increased dramatically since a lot of pairs of samples could be created by a minor percentage of the dataset. Then, considering the rest of the dataset, by 30% of the dataset for the testing process, different fractions (10% to 50%) of the training dataset have been used to train the classifier. A five-fold cross-validation in the data splitting process is used. Then, by using these different fractions of training data, the proposed models have been trained. Two main approaches have been applied to train the classifier (the classifier that includes a retrainable encoder and the classifier that includes an untrainable encoder). In the classifier’s training process, the one that includes an untrainable encoder uses the trained encoder without training it again. The other approach of training the classifier (classifier that includes a retrainable encoder) uses the trained encoder and continues to train the encoder in the classifier with different fractions of the dataset. However, in order to make sure that the results are not affected by having more trainable parameters, another approach for training the classifier has been applied. This approach includes an untrained encoder which makes the classifier have the same number of parameters as the classifier that includes a retrainable encoder. 

## 3. Results and Discussion

### 3.1. Human Activity Recognition Dataset

To show the performance of the model, the dataset of [19] has been used. The CSI dataset collected by them was Wi-Fi-based. In the research [19], by installing the Nexmon tool on a Raspberry pi 4GB, they were able to collect and store CSI data according to the transmitted and received information. They used Raspberry pi 4 and a Tp-link archer c20 as an access point (AP) in 20 MHz, and a personal computer (PC) is used for traffic generation by pinging or watching a movie on the internet. Then the AP will reply with pong packets to the sent pings from the PC. Raspberry pi uses only one pair of transmitters and receivers, but due to its futuristic capabilities and inexpensiveness, it has become a suitable tool for data collection. The Nexmon tool installed on Raspberry pi has also minimized noise by applying filters during data collection, and its configurations were as follows: Core 1, NSS mask 1, 4000 samples, 20 s. The standard used is also IEEE 802.11ac, which is used in 20 MHz bandwidth on channel 36. The AP’s MAC address filter was also set in order to make sure that the Raspberry pi would not connect to another Ap on channel 36. AP and Pi are both located 1m above the ground and they are 3 m away from each other. In the research [19], 4000 CSI samples were collected in the 20 s. After sampling, each line represents 5 ms. The parts related to the activity are completely separated and then the raw CSI is stored in CSV files as matrices with 52 columns (subcarriers) and 600 to 1100 rows (depending on the activity time). Along with the CSI samples, there are labeled files to separate the lines for each activity. Depending on the presented model, the number of samples used for training the model is also different. In other words, depending on the complexity of the work and the selected algorithm, the amount of data required for HAR is different. The dataset used in this research includes seven activities: walk, run, sit down, lie down, stand up, bend, and fall. Each activity was performed 20 times by 3 volunteers of different ages in a room at home. As a result, the dataset includes 420 samples. In this research, only 1.5% of the total data was used for training AE. The data used for AE training is not used for classifier training. In all methods, initially, 20% of the original data are separated for the testing (evaluation) process and different percentages of the remaining data (from 10% to 50% of 80% of the original data) are used for the process of model training, including the validation process. More precisely, from the data which have been separated for the model training, 20% is used for validation, and the remaining data are used for training. The dataset is available on a GitHub repository (https://github.com/parisafm/CSI-HAR-Dataset, accessed on 19 February 2023).

### 3.2. Comparison of Proposed Models

The proposed DL method for classification, along with feature extraction by AE, has shown better performance compared with [19] when we use a fraction of data. In this part, the results of the methods explained in Section 2 are presented and compared. The simulations were performed using the hardware with these specifications: a high-speed GeForce RTX 3070 GPU with 8 GB of memory, 24 GB RAM, and a CPU that is 11th Gen Intel(R) Core (TM) i7-11370H. We used CSI’s domain for simulations. It contains vectors with 52 dimensions (subcarriers). After preprocessing, the dataset is given to the presented models. In all models, 20% of the total data are used for testing, and different percentages of the remaining data are used for training the model; 20% of the training data are also selected for validation in order to be informed about the overfitting. The classifier consists of two CNN layers with the same channel size depicted in Figure 5: as a method for preventing possible overfitting, a dropout of 40% is used in the suggested model; one LSTM layer with a hidden size of 32 and a dropout of 40% are used, as depicted in Figure 5. The dimensions of the convolutional window in both layers are 5 × 5. We used the Adam optimizer with a learning rate of 0.0001. Categorical cross entropy has also been used to obtain loss. The batch size and the number of epochs used in the batch are 64 and 300, respectively.

In the introduced AE, the dimensions of the convolutional window in all layers are considered to be 10 × 10, and the used activation in all convolutional layers is Relu so that the fading problem can be partially covered. Dropout and pooling are not used in the presented AE model. The number of epochs and batch size considered are 50 and 25, respectively. The optimizer used is also Adam, with a learning rate of 0.0001. The size of the window used to convert the CSI data into a multidimensional array is also considered to be 300. The used AE was trained using only 1.5% of the total data, which is a very small amount. This approach is in agreement with the goal of the research, which is using fewer data to achieve high performance. The data used to train AE will not be used for the training of the classifier again. In the three presented methods, AE was used in the classifier in three different manners, and the results were compared with the classifier without AE. In this research, five-fold cross-validation was used to provide more logical and non-random results. While using the five-fold method, the entire training data are not used for model training. In the suggested process, after dividing data into five parts (folds) and selecting four parts for training and validation, and one part for evaluation, from 10% up to 50% of those four parts will be used for further processing. This approach helps using a small size of training data, while the original process of five-fold validation will be kept intact since evaluation data at each fold represents a certain part of full data, and merging evaluation data parts regarding each fold, makes the whole dataset true.

Regarding data with small size (e.g., 10%) and according to Table 1, the classifier without AE outperforms the other models. However, this percentage of the training samples is very small (the same as 20%), and therefore, the performances of the models are not reliable. Therefore, the focus is on the performance accuracy of methods using 50% of the used data. Note that simulations show that using up to 100% of data does not significantly improve the accuracy. According to Table 1, Model c demonstrates better performance than Model a in every fraction of data, excluding 10%. This shows using a trained encoder can perform quite well. Although the number of total parameters varies, in the case of this encoder, the number of trainable parameters is almost the same as in Model a (shown in Table 2), but they do not have the same computational cost since the untrainable encoder layers include their own pretrained parameters, which affect both offline and online phases in Model c. Regarding the online phase and based on the results of Table 3, the little difference between the required time could be ignored due to its great performance.

Model b, which shows the best performance in comparison with other models, is a combination of both methods in Section 2.5.3 and Section 2.5.4. In order to justify that its superior performance is not due to the high computational cost of its training process, Model d is introduced. The comparison between Models b and d demonstrates that, although they have equivalent numbers of trainable parameters (Table 2), the performance is quite different, as shown in Table 1. While Model b achieved better performance (compared to Model c), it has more trainable parameters, which leads to more computational cost and more processing time, according to Table 2 and Table 3, specifically in the offline phase. Hence, Model c is more efficient in the case of the training process because of having a lower computational cost and better performance than Model a. Moreover, as is shown in Table 3, The time consumption during the online phase remains similar across proposed models, including Models c, b, and d.

In comparing Models a and c using 20%, 30%, 40%, and 50% of the data, Model c showed improvement of 5.31%, 12.14%, 9.77%, and 8.7% respectively. By using only 1.5% of the total data for training the AE and 50% of the used data for training the classifier, Model c reached an accuracy of 81.72%. Meanwhile, Model b outperforms Model d when using 20%, 30%, 40%, and 50% of the data, with improvements of 22.21%, 14.69%, 11.74%, and 21.72%, respectively. The best performance seen in this experiment was achieved by Model b with 94.49% accuracy while using 50% of the data. The experimental range of data used in the research was 10% up to 80%, but after reaching 50%, no significant improvement was observed. Therefore, the research aims to use a range of 10% up to 50% of the used data. For example, by using 10% of the data, Model b achieved 37% accuracy, and by using 30% and 50%, 87.27% and 94.49%, respectively. However, beyond 60% up to 100%, the improvement was negligible, with accuracies between 96.8% and 97.5%. Compared to the basic classifier, the biggest improvements have been achieved in the case of using 30% of training data, which are 12.14% and 24.33%, regarding untrainable encoder and retrainable encoder, respectively.

**Figure 12:**
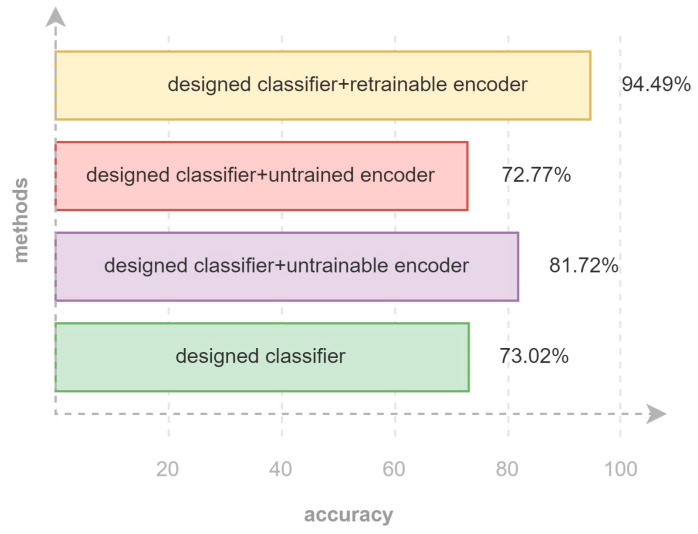
Accuracy of different methods that include MIMO-AE and without autoencoder, implemented on 50% of the training dataset.

**Figure 13:**
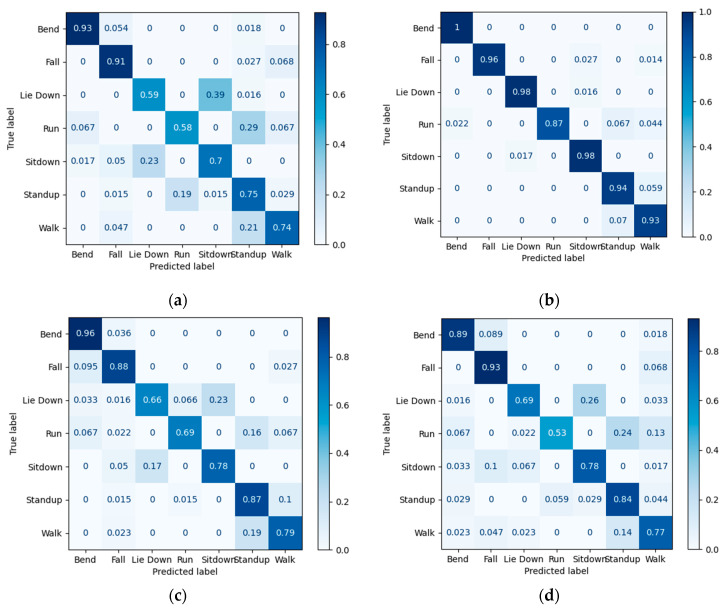
Confusion matrices of proposed methods, using 50% of the used data (including training and validation data): (**a**) designed classifier; (**b**) using retrainable encoder; (**c**) using untrainable encoder; (**d**) using the untrained encoder.

As is shown in Figure 12, using 50% of the used data, the model that includes a retrainable encoder outperforms other training approaches. Although the model includes an untrainable encoder, is known as an efficient approach for training, and is second place in accuracy still, its result is way better than the designed classifier (without the encoder). The confusion matrices presented in Figure 13 have also been used to show the accuracy of four models that were trained with 50% of the used data (including 80% of the total dataset for training and validation) and tested with 20% of the total data and detailed recognition results of each class are presented using confusion matrices in Figure 13. Based on these results, for the activities of sit down and lie down, due to the similar start position and different end of the activity, we expected difficulty in distinguishing these two activities from each other using a small part of data for training the model. However, Model b recognizes the differences between these two activities with high accuracy. To justify that this result is not reached just because of the number of total parameters, a comparison between Models b and d is made. In Model b, out of 20% of the data that were separated for the test, just 1.65% of the sit-down and lie-down data were not distinguished from each other; while in Model d, 16.35% of the test data for these two were wrongly detected. According to matrices, bend, sit down, and lie down activities are detected impressively (100%, 98%, and 98% accuracy, respectively), and detecting run activity is performed with less accuracy (87%). Furthermore, it is crucial to detect falls in this research, as it is relevant to caring for the elderly. According to the confusion matrices of method in Section 2.5.2, this activity has been detected with 96% accuracy using 50% of the used data, which is more than the method in Section 2.5.4. The accuracy of fall events has made this research practical using only a small amount of data.

Due to their fast physical changes in small windows of time, discriminating between running and standing up could be a challenge. As shown in matrices b, 3.35% of the test data could not be discriminated, and as shown in matrices d, 14.95% of the test data for these two activities has not been detected. Therefore, it seems that method in Section 2.5.2 is performed better not just because of the number of parameters but also because the used encoder was trained before. Moreover, for the lie-down and sit-down activities, Model c could not distinguish 20% of these activities in test data and in Model a, there is a 31% error rate. For standing up and running activities, Model c could not detect 8.75% of these activities in test data, and Model a is not able to detect 24% of these activities. As a result, using a pretrained encoder could improve our classifier in order to detect activities. Finally, according to Figure 13, Model b, compared with Model c could achieve performed results in detecting all seven activities.

Two of the proposed methods show the best results compared with others. Therefore, in order to show the performances with different parameters, R2 score, F1 score, precision, and recall have been illustrated in Table 4. All results are based on one-time simulations.

In early trials, simple AEs were considered a competitor for MIMO-AE. However, since the use of fewer data samples is viewed as an advantage, in the case of simple AE, using 1.5% of the data only provides 179 samples for the training process, while comparing to the same situation about MIMO-AE, where the training process has access to 32,041 pairs of samples (179 × 179). This circumstance led to simple AEs, in the best scenario, not properly being trained and, in some scenarios, reaching the overfitting point. To clarify the full aspects of using simple AE, the results of using this AE in the training process of the suggested classifier are presented in Table 5. The structure of this AE is similar to Figure 2, while the only difference is the number of inputs and outputs. As results show, compared to the suggested MIMO-AE, while in the trainable scenario, there is only a 3% difference between best results (in the case of 50% of used data), in the untrainable scenario, this difference increases up to 15%. Therefore, not only this AE fails to reach a similar performance in the case of using it in the trainable model, but in the case of using it as an untrainable model, results are significantly weaker compared to MIMO-AE, and this keeps the complete model from reaching its full potential. Therefore, despite its need for more computational cost, final results justify the training of MIMO-AE since its results are superior compared to simple AEs.

Another examined AE structure in this study for learning latent representation is Variational Autoencoder (VAE). In this autoencoder, instead of encoding an input as a single point, we encode it as a distribution over the latent space [32]. Early simulations showed that this structure could not be trained at all in the case of two-input two-output mode, and in the single-input single-output mode during training, it demonstrates a completely unfavorable behavior in the amount of error function.

In this research, despite the imbalance in the samples of each class, the solving class imbalance methods have not been used. The Synthetic Minority Oversampling (SMOTE) technique leads to balance classes. SMOTE is a statical method that can increase the number of the samples of the classes by generating from existing minority samples. In the first steps of this research, the use of the SMOTE technique [33] was considered a comprehensive and strong solution to solve the class imbalance problem; however, the results demonstrated that there is no need to use this method. The reason for not using this method is due to the fact that in the obtained results, none of the complications of the imbalance class were observed.

### 3.3. Comparing with Others

After considering the method in Section 2.5.2 as the named method presented in this research, it has been compared with the results of [19] as a benchmark. Considering the various challenges of recording HAR-related data, it is more favorable to record a small number of samples, with regard to special conditions of one study, and adjust available data on the web to use them alongside newly recorded samples. Accordingly, the performance of Model b, with respect to the fraction of used data for the training and validation process, has been compared with [19].

In [19], the data considered for the test include 20% of the total data. Then, from the remaining 80% of the data for training and validation, 10% to 50% is selected. As shown in Table 6, regarding 10% of used data, the proposed method has lower accuracy. Since the percentage of used data for training and validation is very small, the obtained results are not reliable. In 20% of the used data, all models performed almost the same. In 30% up to 50% of the used data, the accuracy of the proposed model has improved significantly. To check the performance of the model presented by [19] in detecting each activity, the confusion matrix has been used. Two methods from [19] have been selected for comparison with the best method presented in this research. One of these methods was classification using 2D-CNN, which had the highest accuracy among the rest of the methods presented in [19], and the other was classification using LSTM, which is expected to perform well for time series data. According to Table 6, the proposed model in this research, in the case of 30% of used data, has an 18.89% improvement compared to 2D-CNN and 9.01% improvement compared to LSTM. In 40% of the used data, 22.96% and 18.57% improvements have been shown using the proposed method in this research compared to 2D-CNN and LSTM, respectively. In 50% of the used data, using the proposed model instead of 2D-CNN and LSTM for detection has improved performance by 21.94% and 17.77%, respectively.

**Figure 14:**
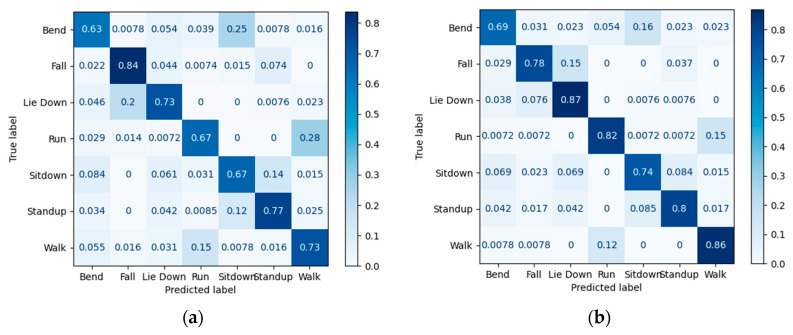
Confusion matrices of previous methods using 50% of the used data (including 80% of total data for training and validation data): (**a**) 2D-CNN [19]; (**b**) LSTM [19].

Therefore, when the entire available data is not used for training, the presented models in [19] do not show remarkable performance compared to the proposed method in this research. This comparison can be made in detail strictly in the confusion matrix (Figure 14). The performance accuracy of the presented matrices (Figure 14) is only based on using 50% of the used data for model training. Testing the models and obtaining accuracies have also been done using only 20% of the total data. Since walking and running share great similarities, regarding people’s physical behavior, we compare the activity of walking and running using the confusion matrix. In Model b, 2.2% of the samples of these two activities are misdiagnosed. While in the 2D-CNN and LSTM models presented in [19], 21.5% and 13.5% of the samples of these two activities are wrongly recognized, respectively. Both sit-down and lie-down activities, which had the same start position, have been perused in the confusion matrices (Figure 14). Accordingly, in the Model b presented in this research, only 1.65% of the test samples were not distinguished from each other, and in the 2D_CNN and LSTM models of [19], 3.05% and 3.83% of the samples were not distinguishable, respectively. As a result, these two activities can be detected from each other to a good extent in all three methods. However, based on the method of Section 2.5.2, the accuracy of discriminating sit-down and lie-down activity samples is generally higher than similar results in [19]. In this research, the accuracy obtained from both sit-down and lie-down was 98%, respectively, while in [19] research, the accuracy obtained from them was 67% and 73% in 2D-CNN, and 74% and 87% in LSTM. In addition, the accuracy for bend activity in our proposed method is 100%, but in other methods in [19], the accuracy is quite lower. For the most part, the method presented in this research becomes particularly important due to the lack of human-centered data because method of Section 2.5.2 has achieved 94.49% accuracy by using only 50% of the used data (which is 80% of the total dataset). Therefore, the extraction of similarities by the trained encoder in MIMO-AE using only 1.5% of total data and the fine-tuning process has proven to become quite effective.

Furthermore, in [19], after converting CSI data into image-like arrays and classification using 2D-CNN, despite the use of the entire data, the accuracy of the model performance reached 95% (the best result among the rest of the [19] methods), while we have achieved 94.49% accuracy by using the retrainable encoder by only 50% of used CSI data.

## 4. Conclusions

This study aimed to train effective HAR models while focusing on using a limited number of samples. To do so, the idea of transfer learning has been utilized. By doing so, prior knowledge of the pretrained encoder, which is part of a larger two-input two-output autoencoder, is used as a subnetwork of designed classifiers. This idea is executed in three different manners, using the encoder as a retrainable, untrainable, and untrained network. The results of these three classifiers have been compared with counterparts in a fair condition. The final results demonstrated that in order to present a model with high evaluation accuracy, it is not necessary to provide the training process with a huge chunk of data, and it is more efficient to extract features and information from them with a more efficient process. Regardless of aiming for higher accuracy or a more beneficial training process, MIMO-AE provides a noticeable improvement in the performance of the classifier. Compared to the basic classifier, the improvements that have been achieved in the case of using 50% of training data are 8.7% and 21.47%, regarding the untrainable encoder and retrainable encoder, respectively. They achieved 81.72% and 94.49% accuracy. While this study uses CSI as its main representation of data, for future work, it is possible to use extra steps, such as wavelet and spectrogram transforms, for better frequential representation. Moreover, the structure of multi-input multi-output autoencoders can be redesigned using other possible options. In addition, a better network could be introduced for the autoencoder in order to achieve better performance while using an untrainable encoder in the classifier.

## Figures and Tables

**Table 1:** Average accuracy comparison of proposed models using different percentages of data in case of using MIMO-AE.

Percentage of Used Dataset for Training and Validation	Retrainable Encoder + Classifier (Model b)	Untrained Encoder + Classifier (Model d)	Untrainable Encoder + Classifier (Model c)	Designed Classifier (without Encoder) (Model a)
10%	37	20.98	39.79	39.90
20%	68.2	45.99	62.65	57.34
30%	87.27	72.58	75.08	62.94
40%	93.21	81.47	79.3	69.53
50%	94.49	72.77	81.72	73.02
60%	93.5	91.65	83.22	80.02
70%	95.87	81.94	87.27	81
80%	96.75	94.6	87.96	80.5

**Table 2:** The total number of trained and untrained parameters.

	Retrainable Encoder + Classifier (Model b)	Untrained Encoder + Classifier (Model d)	Untrainable Encoder + Classifier (Model c)	Designed Classifier (without Encoder) (Model a)
Total parameters	2,227,131	2,227,131	2,227,131	11,659
Trained parameters	2,227,107	2,227,107	12,235	11,635
Untrained Parameters	24	24	2,214,896	24

**Table 3:** The process time (sec) for retrainable encoder, untrained encoder, and designed classifier (without encoder) methods.

Percentage of Used Dataset for Training and Validation	Retrainable Encoder + Classifier (Model b) (Train/Test)	Untrainable Encoder + Classifier (Model c) (Train/Test)	Designed Classifier (without Encoder) (Model a) (Train/Test)
10%	512.27, 7.62	210.22, 7.58	54.05, 0.6
20%	854.6, 1.03	284.14, 1.03	44.0, 0.46
30%	1225.54, 1.23	336.28, 1.03	49.37, 0.52
40%	1593.31, 1.06	431.77, 1.4	59.47, 0.52
50%	1951.65, 1.11	515.32, 1.14	64.04, 0.44

**Table 4:** A table of methods including an untrainable encoder and retrainable encoder’s R2 score, F1 score, precision, and recall.

Percentage of Used Dataset for Training and Validation	R2 Score (Retrainable/Untrainable)	F1 Score (Retrainable/Untrainable)	Precision (Retrainable/Untrainable)	Recall (Retrainable/Untrainable)
10%	0.1, -0.42	0.33, 0.33	49.7, 41.74	38.08, 38.08
20%	0.62, 0.23	0.65, 0.59	71.12, 66.25	65.84, 61.91
30%	0.84, 0.48	0.88, 0.68	88.43, 68.82	88.45, 68.30
40%	0.89, 0.7	0.92, 0.8	92.33, 80.12	92.13, 80.58
50%	0.9, 0.69	0.94, 0.8	94.6, 82.19	94.59, 81.08

**Table 5:** Average accuracy comparison of proposed models using different percentages of data, in the case of simple AE versus MIMO-AE.

Percentage of Used Dataset for Training and Validation	Retrainable Encoder + Classifier (Simple AE)	Untrainable Encoder + Classifier (Simple AE)	Retrainable Encoder + Classifier (MIMO-AE)	Untrainable Encoder + Classifier (MIMO-AE)
10%	35.87	31.7	37	39.79
20%	64.37	48.16	68.2	62.65
30%	84.52	53.56	87.27	75.08
40%	91.4	52.09	93.21	79.3
50%	92.32	66.34	94.49	81.72
60%	91.56	68.3	93.5	83.22
70%	93.33	65.6	95.87	87.27
80%	94.08	77.89	96.75	87.96

**Table 6:** Comparing average accuracy of classifier included by the retrainable encoder with LSTM and 2D-CNN-based classifiers from [19] using different percentages of data.

Percentage of Used Dataset for Training and Validation	LSTM [19]	2D-CNN [19]	Retrainable Encoder + Classifier (Model b)
10%	64.76	55.54	37
20%	68.27	62.23	68.2
30%	78.26	68.38	87.27
40%	74.64	70.25	93.21
50%	76.72	72.55	94.49

## Data Availability

The dataset used in this study can be downloaded at: https://github.com/parisafm/CSI-HAR-Dataset (accessed on 19 February 2023).
